# Serum-Free Medium Optimization Based on Trial Design and Support Vector Regression

**DOI:** 10.1155/2014/269305

**Published:** 2014-10-14

**Authors:** Jian Xu, Fang-rong Yan, Zhi-hui Li, Deng Wang, Hai-lin Sheng, Yu Liu

**Affiliations:** ^1^State Key Laboratory of Natural Medicines, School of Life Science and Technology, China Pharmaceutical University, 24 Tong Jia Xiang, Nanjing 210009, China; ^2^College of Science, China Pharmaceutical University, 24 TongJia Xiang, Nanjing 210009, China; ^3^Research Center of Biostatistics and Computational Pharmacy, 24 Tong Jia Xiang, Nanjing 210009, China

## Abstract

The Plackett-Burman design and support vector machine (SVM) were reported to be used on many fields such as some feature selections, protein structure prediction, or forecasting of other situations. Here, with suspension adapted Chinese hamster ovary (CHO) cells as the object of study, a serum-free medium for the culture of CHO cells in suspension was optimized by this method. Support vector machine based on genetic algorithm was used to predict the growth rate of CHO and prove the results from the trial designs. Experimental results indicated that ZnSO_4_, transferrin, and bovine serum albumin (BSA) were important ones. The same conclusion was arrived at when the support vector regression model analyzed the experimental results. With the methods mentioned, the influence of 7 medium supplements on the growth of CHO cells in suspension was evaluated efficiently.

## 1. Introduction

Chinese hamster ovary (CHO) cells are the most widely used mammalian cells in the world for expression and large-scale recombinant protein production. CHO cells can provide stable and accurate glycosylation; they offer a posttranslationally modified product and thus a more accurate in vitro rendition of the natural protein. CHO cells are ideal hosts of different complex biological macromolecules. In order to improve the productivity, optimization of cell culture medium to meet the nutrient demands and to minimize toxic production in cell culture has played a critical role [1–3].

Serum, used to be the most important supplement in the traditional medium for the culture of mammalian cells, provides essential nutrients, hormones, peptides, growth factors, adhesion factors, and so on. However, serum has many shortages such as high cost, batch variability, interference with the downstream processing of product purification, and the desirable elimination from the culture process, for example, viruses, mycoplasma, or prions [4]. Therefore, several commercial serum-free media for CHO cells have been available. However, the compositions of them are proprietary, and the prices of them are expensive. Since, different cell lines of CHO have different nutrient requirements, and no universal serum-free media applicable to all cell lines are available. Thus, a specific serum-free medium for each individual cell line needs to be developed to maximize cell growth. It is necessary for us to develop new serum-free medium used to express a kind of anti-VEGF monoclonal antibody by CHO [5].

However, the nutrients and their interactions which account for the proliferation effects on mammalian cells are complex. It is a time-consuming work and has the risk of neglecting interactions among supplements [6]. More and more statistical designs have been introduced to reduce the time and labors. Chun et al. [7] optimized the serum-free medium by the application of factorial design. Petiot et al. [8], by using fractional factorial design to screen serum-free medium rapidly for the growth of adherent Vero cells, enhanced the concentration of Vero cells to 160%. Deshpande et al. [9] used central composite design to optimize the concentrations of glucose, glutamine, and inorganic salts for cultivation of a CHO cell line.

In this study, a Plackett-Burman statistical design, which provides an efficient way of screening a large number of medium supplements combination, was used to develop serum-free medium. For the sake of recognizing the major supplements in the medium as well as predicting the growth rate, support vector machine (SVM) was also employed. In this paper, we designed the experiments with Plackett-Burman design which can decrease the cost of time and expenses and response surface design which gives a clear response. SVM was introduced to predict the growth rate and to find out the major elements which have a significant influence on the CHO growth rate. These will be described in Section 2. In Section 3, results will be presented to prove our method worked well. In the end, conclusions are drawn in Section 4.

## 2. Materials and Methods

### 2.1. Cell Line and Cell Culture

CHO-K1 cells, obtained from ATCC, were initially maintained in DMEM/F12 (a mixture of DMEM and F12) supplemented with 10% newborn bovine serum at 37°C in the presence of 5% CO_2_. Then, the cells were adapted to serum-free medium by stepwise decreasing the serum concentration by 50% every three passages until the serum concentration was reduced below 0.1%. These cells adapted to the serum-free medium were cultured for further use.

### 2.2. Experimental Design

We applied the Plackett-Burman design accounting for its advantages to find out the main factors having influence on the experiment. It takes two levels of each factor for analysis and a significant factor to be determined by comparison between the two levels of the various factors and the overall difference. Screening tests cannot distinguish between the interaction and the main effects, but a significant effect on the factor can be determined, so as to achieve the purpose of screening. It can be avoided that too many factors or fractional factorial experiment was not significant, wasting resources in the optimization test afterward.

Based on the results of previous experiments and literature review, 7 medium supplements including ZnSO_4_, transferrin, putrescine, bovine serum albumin (BSA), ferric citrate, sodium pyruvate, and ethanol amine were selected as factors to evaluate their stimulatory effects on CHO cells under serum-free conditions [10–13]. Every factor has two levels of concentration: high concentration (+1) and low concentration (−1), given in Table 1. The data were presented from triplicate experiments. Results are considered significant at a* P* value <0.05.

Experiments, based on a Plackett-Burman design, were performed in triplicate in 12-well tissue culture plates containing 1 mL of serum-free media and medium supplements. CHO cells were inoculated at a concentration of 10^5^ cells/mL. The cell concentration and viability were determined by the Trypan blue exclusion method after 72 h of culture.

### 2.3. Support Vector Regression Model

Support vector classification (SVC) and support vector regression (SVR) are the two main categories for SVM. We established the SVR model for prediction, since we tend to get the growth rate of CHO with 7 medium supplements and to recognize the most important components of them. With the established model, we can not only forecast the specific growth rate given factor levels but also pick out the main factors which influence the response values significantly. The SVR builds models depending on a subset of training data, because the cost function for building the model ignores any training data that is close to the model prediction which makes the SVM for regression possible and efficient [14]. As the nutrients and the interactions of them affecting the growth of CHO cells are complex, SVR nonlinear property is much more proper than the conventional linear regression for our problem, in particular the relationship among the elements in the serum-free medium.

The basic idea in SVM is mapping *x* input into a higher dimensional feature space via a nonlinear mapping and accomplishing linear regression in this space [15]. Therefore, regression approximation addresses the problem of estimating a function based on a given dataset *G* = {(*x*_*i*_,*y*_*i*_)^*T*^}_*i*_^*n*^, where *x*_*i*_ ∈ *R* ^*m*^ is the input vector, *y*_*i*_ ∈ *R* ^1^ is the targeted output, *m* is the dimension of the input vector, and *n* is the total number of samples. The standard form of support vector regression [16, 17] is

(1) min⁡w,b,ξ,ξ∗   12||w||2+C∑i=1nξi+C∑i=1nξi∗subject  to   wTϕ(xi)+b−yi≤ε+ξi     yi−wTϕ(xi)−b≤ε+ξi∗     ξi,ξi∗≥0, i=1,2,…,n,

where *ε* is called the tube size of SVM and *C* is the regularization constant determining the tradeoff between the empirical error and the regularized term. Utilizing the Lagrange multipliers, dualization becomes

(2) min⁡α,α∗    12(α−α∗)TQ(α−α∗)+ε∑i=1n(αi+αi∗)       +∑i=1n(αi+αi∗)yisubject  to  ∑i=1n(αi−αi∗)=0, 0≤αi,      αi∗≤C, i=1,…,n,

where *Q*_*ij*_ = *K*(*x*_*i*_, *x*_*j*_) ≡ *ϕ*(*x*_*i*_)^*T*^ *ϕ*(*x*_*j*_), *K*(*x*_*i*_, *x*_*j*_) is the kernel function, *ϕ*(*x*) is the high dimensional feature space, which is nonlinearly mapped from the input space *x*, and *α*_*i*_ and *α*_i_^*^ are the introduced Lagrange multipliers. In above *ε*-SV regression, the goal is to find a function *f*(*x*) which has the most *ε* deviation from the actually obtained targets *y*_*i*_ for all the training data and as flat as possible. The standard SVR to solve the approximation problem is described as the following form:

(3) $$\begin{array}{c} f\left(x\right)=\overset{N}{\underset{i=1}{\sum }}\left(\alpha^{*}-\alpha_{i}\right)K\left(x,x_{i}\right)+b. \end{array}$$

After the models are trained by solving the above optimization problems, we evaluate the predictions by outputs mean squared error (MSE):

(4) $$\begin{array}{c} \text{MSE}=\frac{1}{2}\overset{n}{\underset{i=1}{\sum }}{\left(f\left(x_{i}\right)-y_{i}\right)}^{2}. \end{array}$$

In this study, we considered 12 samples consisting of the different level combinations of seven supplements. Each variable stands for an element, and the response is the specific growth rate of cells. The radial basis function (RBF) kernel $K\left({\overset{-}{x}}_{i},{\overset{-}{x}}_{j}\right)=\exp\left(-\gamma {||{\overset{-}{x}}_{i}-{\overset{-}{x}}_{j}||}^{2}\right)$ was chosen to build the SVR regression. As a result, the *γ* parameter must be an appropriate value which significantly influences the prediction accuracy. Based on the above foundation above, it can be implemented as follows to realize the SVR: data scaling, model training, and model prediction. When training the model, parameter choosing seems to play a momentous role, so we illustrate this in the next section in detail. The model was established through LIBSVM, Matlab version 7.10 [18].

### 2.4. Genetic Algorithm for Parameter Selection

SVM is simplified greatly because of kernel function rather than calculating the complicated nonlinear transformation directly. RBF kernel function was used in this study, so kernel parameter *γ* must be chosen properly to make the SVM model a well-performed one. The penalized parameter *c*, balancing the empirical error and the regularized term, has an influence on the regression model. Plenty of methods have been explored in the parameter selection, such as grid search, particle swarm optimization, and genetic algorithm (GA). GA was employed in our SVM parameter selection for the sake of searching for appropriate parameters in a larger range. GA is a simulation of the biological evolution process in Darwin's natural selection and genetics. It is composed of three basic operations, namely, selection, crossover, and mutation. The parameter selection process is carried out according to the following schema. First, select two original parameters, called parents, and apply the crossover to create two new solutions which are muted (with a given probability). Second, replace the parents by their offspring, new parameters. These two actions repeat for a fixed number of times, so-called generation. Finally, the selected individuals are copied backed to the population to replace the worst ones [19]. These steps repeat until the most suitable parameters are chosen [20].

### 2.5. Feature Selection

Since we focus on the main supplements in the medium, with the SVR model above, this purpose was reached with mean impact value (MIV) which was proposed by Dombi reflecting the change of weighed matrix in SVM model. We implemented the idea in our study and build MIV-SVR variable selection method. MIV is used to find out the most influential variables to the SVR model. Its absolute value shows the relative importance of corresponding variable in the model. Before searching for the importance, SVR model should be trained well. Then, plus and minus 10% to each variable in the train dataset to make it into two new datasets. New datasets are applied as simulation samples to the model and consequently simulation results are obtained. The difference between the new outcomes is MIV which demonstrates the impact to the outputs.

## 3. Results and Discussion

### 3.1. Plackett-Burman Design

The nutrients and the interactions of them affecting the growth of CHO cells are complex. To find the key factors, the Plackett-Burman design was used to achieve it. It is a statistical design providing an efficient way of screening a large number of medium supplements and identifying the important factors.

The two-level Plackett-Burman design was performed using seven medium supplements. For purpose of modeling, a value of “1” was assigned to those with high concentrations, and a value of “−1” was assigned to those with low concentrations. We considered the specific growth rate as the response value obtained from the experiments. The design of Plackett-Burman and the response values are shown in Table 2. From the analysis of range, given in Table 3, the ranges of *X*_1_, *X*_4_, and *X*_5_ are larger. *T*1 and *T*2 are the sum of each factor's results in level 1 and level −1, while *t*1 and *t*2 represent the mean value. In addition, *R* is the range of *t*1 and *t*2. It suggests that ZnSO_4_ (*X*_1_), BSA (*X*_4_), and Ferric citrate (*X*_5_) were important ones influencing the growth of CHO cells. The results of the analysis of variance, given in Table 4, also showed that those factors were the most important ones.

### 3.2. The Response Surface Design

**Figure 1:**
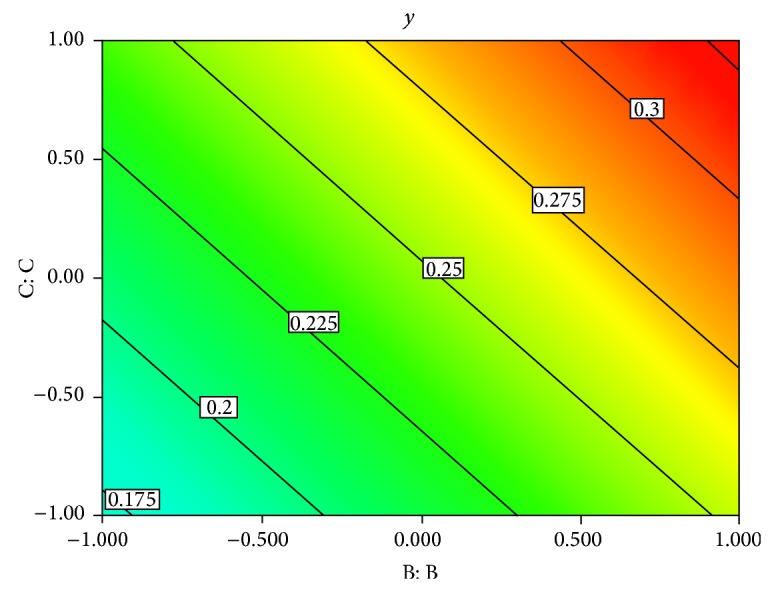
Specific growth rate contour map of BSA (B) and Ferric citrate (C).

**Figure 2:**
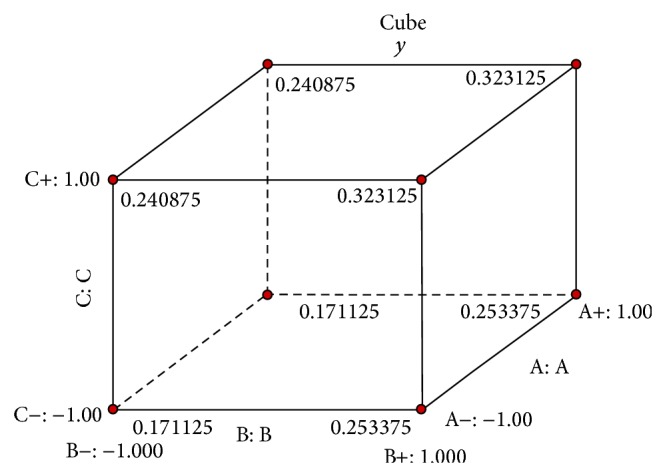
Cube combination of ZnSO_4 _(A), BSA (B), and Ferric citrate (C).

Contour line in Figure 1 represents the growth rate value varying from 0.124 to 0.319, *y* is the growth rate, and the color in the contour means the difference value region. Only BSA (*X*_4_) and Ferric citrate (*X*_5_) showed significance in ANOVA although ZnSO_4_ (*X*_1_) was also chosen as one of the important elements. That is because the *P* value of *X*_1_ was 0.0589, approaching to 0.05 when all the seven elements are concerned. Moreover, SVM regression model showed the MIV of *X*_1_ ranked third which is in accordance with the ANOVA results. Design-Expert also gives three elements cube design results that if ZnSO_4_ (*X*_1_), BSA (*X*_4_), and Ferric citrate (*X*_5_) are given level 1, it can reach the largest response 0.3231, shown in Figure 2.

### 3.3. The SVR Model and Key Factors

**Figure 3:**
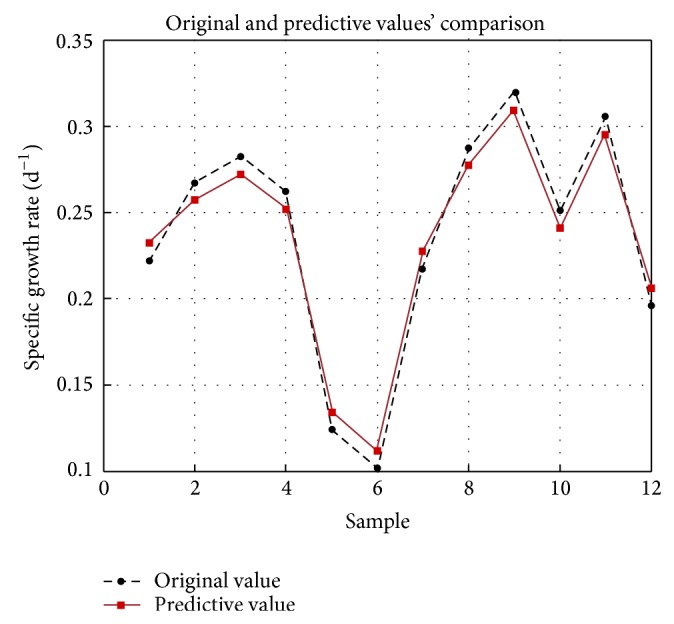
Original and predictive value of specific growth rate.

The parameters *c* = 2.3677 and *γ* = 91.9133 with the GA parameters terminal generation 200 and population size 20. With these parameters, the SVR model is well performed with a MSE = 0.0045943 and *R* ^2^ = 0.9808. As shown in Figure 3, the original data and predicted values are rather closed. That is to say, the SVR model can predict the growth rate accurately. Although the Plackett-Burman design picked out key factors, MIV values in SVR model proved the result as well. We selected three important components, namely, Ferric citrate (*X*_5_), BSA (*X*_4_), and ZnSO_4_ (*X*_1_). This result was consistent with the Plackett-Burman design.

### 3.4. Discussion

The Plackett-Burman design and support vector machine (SVM) make the multifactor experiments consume less time, materials, and labor. Experimental designs, such as uniform design, orthogonal design, and Plackett-Burman, not only reduce the times testing but also predict the optimal case. Mathematical modeling is usually taken into account when dealing with the experimental data. However, the limit of the time and other factors, there cannot be a lot of times testing different combined factors in different levels. It makes the data analysis troublesome, needing alternative modeling methods based on the small sample. The SVM is the data modeling tool which is designed specifically for small sample analysis. As for this experiment, the main goal is to find the optimal factorial composition, so the SVM make it possible to predict, optimize, and figure out the best parameters in the case.

As shown in Table 2, the Plackett-Burman design samples and corresponding response were obtained. The cost in experiments has been decreased through reasonable trial design. However, taking SVR modeling into account, the sample size maybe was a little small. Because the trial design covered level combinations needed, all the data were used to train and predict can be a practical strategy. Moreover, two levels were set based on the literature, whether more levels can give different performances to be studied.

As the cell line used here produces a recombinant antibody, it would be important to see the effects that these components have on production. But, the serum in medium will interfere with the antibody measuring. Although cell growth does not always correlate with production, for this CHO variant, growth was shown to be proportional to antibody production in our previous test.

**Figure 4:**
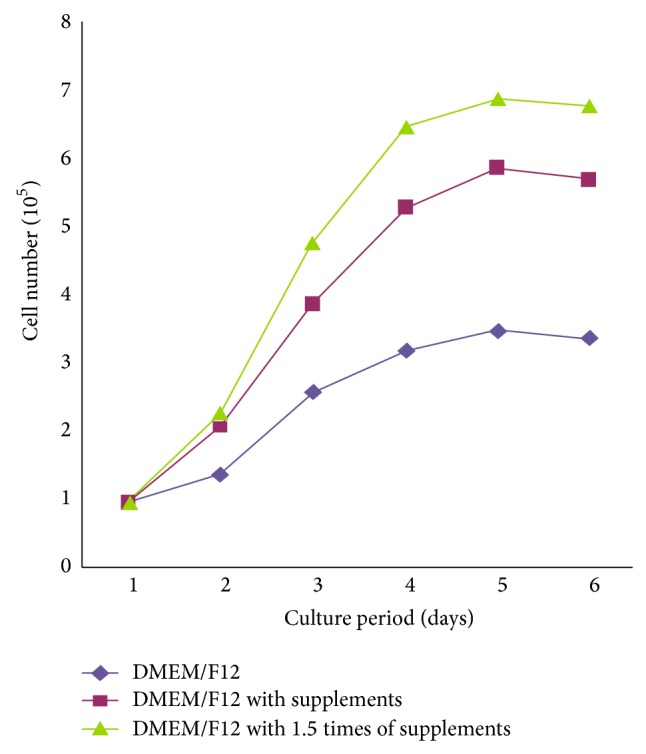
Growth rate after being supplied with 1.5 times supplements.

We also noted that the optimized parameters (red area) were focusing on the edge of the contour line Figure 1. That's mean maybe the level range we chose before was not wide enough to get the greatest growth rate. So, another experiment was taken to see whether the growth rate could be raised after being cultured with 1.5 times supplements. Results are shown in Figure 4, and the growth rate was raised after enlarging the level range.

## 4. Conclusions

In this work, we employed Plackett-Burman design and SVM to explore the influence of 7 medium supplements on the growth of CHO cells in suspension. Results suggested that statistical design methodology offered an efficient and feasible approach for cells growth optimization. This method showed efficient and reliable property when the SVM gave the same result. It is a powerful technique in reaching an optimized solution and accurately analyzing the interactive effects of most influential medium supplements. Furthermore, SVR was introduced to the analysis of supplements on CHO cells growth, and it can predict the growth rate for new sample based on the training data. It is easy and appropriate using GA to choose suitable parameters which play momentous role in SVR model. With SVM, trial design, and other statistical methods, experiments for CHO cells become simplified and credible.

## Figures and Tables

**Table 1:** Range of different factors investigated with Plackett-Burman design.

Factors	Level	
	−1	1
*X*_1_ (ZnSO_4_)	0.4 mg/L	1 mg/L
*X*_2_ (transferrin)	4 mg/L	10 mg/L
*X*_3_ (putrescine)	0.4 mg/L	1 mg/L
*X*_4_ (BSA)	80 mg/L	200 mg/L
*X*_5_ (ferric citrate)	0.3 mmol/L	1 mmol/L
*X*_6_ (sodium pyruvate)	0.3 mmol/L	1 mmol/L
*X*_7_ (ethanol amine)	2 mg/L	6 mg/L

**Table 2:** Matrix of Plackett-Burman design and response values.

Trial	*X*_1_	*X*_2_	*X*_3_	*X*_4_	*X*_5_	*X*_6_	*X*_7_	Specific growth rate (d^−1^)
1	1	1	−1	1	−1	−1	−1	0.222
2	−1	1	1	1	−1	1	1	0.267
3	−1	−1	−1	1	1	1	−1	0.282
4	1	−1	1	1	−1	1	−1	0.262
5	−1	−1	−1	−1	−1	−1	−1	0.124
6	−1	1	−1	−1	−1	1	1	0.102
7	−1	1	1	−1	1	−1	−1	0.217
8	1	−1	−1	−1	1	1	1	0.287
9	1	1	−1	1	1	−1	1	0.319
10	1	1	1	−1	1	1	−1	0.251
11	−1	−1	1	1	1	−1	1	0.305
12	1	−1	1	−1	−1	−1	1	0.196

**Table 3:** Analysis of range.

	*X*_1_	*X*_2_	*X*_3_	*X*_4_	*X*_5_	*X*_6_	*X*_7_
*T*1	0.512	0.46	0.5	0.552	0.554	0.484	0.492
*T*2	0.432	0.486	0.446	0.392	0.392	0.462	0.452
*t*1	0.256	0.23	0.25	0.276	0.277	0.242	0.246
*t*2	0.216	0.243	0.223	0.196	0.196	0.231	0.226
*R*	0.04	0.013	0.027	0.08	0.081	0.011	0.02

**Table 4:** Analysis of variance and response values significance.

Source	DF	Anova SS	Mean square	*F* value	*Pr*⁡>*F*
*X*_1_	1	0.0048	0.0048	6.86	0.0589
*X*_2_	1	0.0005	0.0005	0.72	0.4427
*X*_3_	1	0.0022	0.0022	3.12	0.1519
*X*_4_	1	0.0192	0.0192	27.42	0.0064
*X*_5_	1	0.0198	0.0198	28.34	0.0060
*X*_6_	1	0.0004	0.0004	0.55	0.4994
*X*_7_	1	0.0012	0.0012	1.66	0.2674
